# Visualization and quantification of dynamic intercellular coupling in human embryonic stem cells using single cell sonoporation

**DOI:** 10.1038/s41598-020-75347-4

**Published:** 2020-10-26

**Authors:** Zhenzhen Fan, Xufeng Xue, Jianping Fu, Cheri X. Deng

**Affiliations:** 1 Department of Biomedical Engineering, University of Michigan, Ann Arbor, MI 48109 USA; 2 Department of Mechanical Engineering, University of Michigan, Ann Arbor, MI 48109 USA

**Keywords:** Biological techniques, Biophysics, Stem cells

## Abstract

Gap junctions (GJs), which are proteinaceous channels, couple adjacent cells by permitting direct exchange of intracellular molecules with low molecular weights. GJ intercellular communication (GJIC) plays a critical role in regulating behaviors of human embryonic stem cells (hESCs), affecting their proliferation and differentiation. Here we report a novel use of sonoporation that enables single cell intracellular dye loading and dynamic visualization/quantification of GJIC in hESC colonies. By applying a short ultrasound pulse to excite single microbubbles tethered to cell membranes, a transient pore on the cell membrane (sonoporation) is generated which allows intracellular loading of dye molecules and influx of Ca^2+^ into single hESCs. We employ live imaging for continuous visualization of intercellular dye transfer and Ca^2+^ diffusion in hESC colonies. We quantify cell–cell permeability based on dye diffusion using mass transport models. Our results reveal heterogeneous intercellular connectivity and a variety of spatiotemporal characteristics of intercellular Ca^2+^ waves in hESC colonies induced by sonoporation of single cells.

## Introduction

Cell–cell communication plays an essential role in controlling the organization, coordination, and development of multicellular organisms. While diverse mechanisms exist for the exchange of molecular information between cells, the proteinaceous channels between adjacent cells, known as gap junctions (GJs), provide a direct mechanism for the transfer of Ca^2+^ and other molecules of small molecular weight (MW) between neighboring cells^1^. Cell–cell metabolic and electrical coupling are mediated by such GJ intercellular communication (GJIC), which is critical for proper functions of multicellular organisms^2^. For example, loss of direct intercellular communication has been associated with cancer onset and progression^3–5^. GJs have also been found to play a critical role throughout the development of mammalian embryo^6,7^, as the coordinated development of multicellular embryonic tissues requires rapid and robust intercellular communications.

Human embryonic stem cells (hESCs), derived from the inner cell mass of pre-implementation human blastocysts, can differentiate into somatic cells associated with the three germ layers^8^. Isolation and in vitro culture of hESCs have opened new opportunities for studying basic stem cell biology and embryonic development^9–16^. Directed differentiation of hESCs generates specific cell types useful for regenerative medicine, disease modeling, and drug screening^17^. However, improved culture protocols for maintaining and controlled differentiation of hESCs are required for successful use of hESCs in these applications, and it has been recognized that cell–cell interaction during in vitro culture of hESCs remains an intriguing yet incompletely understood feature that emerges on a cell colony scale to mediate important hESC behaviors including pluripotency^18,19^ and community behaviors^20,21^.

By allowing chemical signals^19,22^ and even mechanical effects to operate over multicellular distances, cell–cell communication enables hESCs to sense the presence of each other^23^ and coordinate their differentiation and function^9,10,22,24^. Not surprisingly, functional GJIC has been identified as a common feature of hESC colonies maintained under various culture conditions^25,26^. Since cell–cell communication affects hESC survival and differentiation, improved understanding of GJIC may help identify factors that promote efficient culture conditions of hESCs^27^.

Common techniques for assaying GJIC and its pathophysiological alternations^28^ include scrape loading/dye transfer (SL/DT), microinjection of fluorescent tracers, and paired electrophysiological recordings. In the SL/DT assay^28,29^, mechanically scraping a cell monolayer disrupts the membrane of a large number of cells and allows small fluorescent dye molecules to enter the cytoplasm of live cells through functional GJ adjacent to disrupted cells. After removal of extracellular dye molecules in culture medium, GJIC is studied by examining spatial extent of dye transfer in the remaining live cells using fluorescent microscopy. The SL/DT assay allows assessment of average features of GJIC in a cell monolayer; however, evaluation of GJIC at the single cell level and the dynamic process of cell–cell communication is not feasible^28^. Microinjection of fluorescent tracer into cells^30,31^ offers single cell resolution for assessing GJIC; however, this method is low-throughput and labor intensive, typically requiring manual handling of individual cells. Similarly, paired electrophysiological recordings^32,33^, even though offering a superior temporal resolution, are limited to studying GJIC between a pair of cells. Electroporation, which uses electric pulses to disrupt cell membrane, can load dye molecules into a large number of cells^34^; but suitable electrodes and protocols are needed for single cell operations and minimizing cell detachment/death.

To overcome these limitations, we employed in this study single cell sonoporation as a new method for controlled dye loading and dynamic visualization of cell–cell coupling in hESC colonies. Established as imaging contrast agents for diagnostic ultrasound imaging^35^, microbubbles with stabilizing lipid or polymer shells (radius 1–3 µm) are biocompatible and have also been exploited for non-viral intracellular drug/gene delivery applications^35–38^. When subjected to ultrasound excitation^35,39,40^, microbubbles expand/contract or even collapse, generating localized mechanical impact on cells to induce transient poration of cell membrane (sonoporation)^38,41,42^. In particular, we have shown that single cell sonoporation^38,42^ mediated by acoustic cavitation of individual membrane-anchored microbubbles^41,43^ generated nanoscale, reparable pores on the targeted cells to allow controlled intracellular delivery of membrane impermeable molecules without affecting cell viability.

While sonoporation has been studied extensively for intracellular drug and gene delivery, in this study, we report a novel use of single cell sonoporation for assaying cell–cell communication in hESCs. Sonoporation is used to load fluorescent molecules into single cells and subsequent dynamic molecular coupling between the sonoporated cells and adjacent daughter cells was measured to assess GJIC at the single cell level. In addition, we report the use of single cell sonoporation for the initiation of intercellular Ca^2+^ waves from single cells to study their characteristics in hESC colonies. Conventional methods use endogenous signals or external chemical stimulants such as ATP^44^ to initiate [Ca^2+^]_i_ changes^45^. However, global application of ATP or other chemical agents to cell culture medium was generally without spatial specification, making it difficult to investigate changes in [Ca^2+^]_i_ that propagate from cell to cell in the form of intercellular Ca^2+^ waves. In contrast, in this study, we show that single cell sonoporation enables a bolus influx of Ca^2+^ into single cells, from which intercellular Ca^2+^ waves are initiated and detected with high spatiotemporal resolution^36,46,47^.

## Materials and methods

### Cell culture

Human embryonic stem cell line H9 (WA09, WiCell; NIH registration number: 0062) was cultured in a standard culture system using mTeSR1 medium (Stemcell Technologies) and lactate dehydrogenase-elevating virus (LDEV)-free human embryonic stem cell qualified reduced growth factor basement membrane matrix Geltrex (Thermo Fisher Scientific) per manufacturer instruction. The cell line was test negative for mycoplasma contamination (LookOut Mycoplasma PCR Detection Kit, Sigma-Aldrich). hESCs were seeded as single cells on glass bottom dishes (MatTek Corporation) coated with 1% (v/v) Geltrex at a density of 20,000 cells cm^-1^ with ROCK inhibitor Y27632 (10 µM; Tocris). 24 h after cell seeding, cell culture medium was replaced with fresh mTeSR1 medium without Y27632. Sonoporation experiments were conducted one day after cell seeding (day 1). For other experiments, hESCs were cultured in mTeSR1 medium up to day 8, without losing pluripotency. Culture medium was replenished daily.

### Targeted microbubbles

In order to achieve stable spatial position of microbubbles on the cell surface to generate controlled single cell sonoproation, we functionalized microbubbles with Arg-Gly-Asp (RGD) peptides to attach to cells via RGD-integrin binding. Targestar-SA (Targeson) microbubbles (1 × 10^9^ bubbles/ml) were conjugated at room temperature to biotinylated Arg-Gly-Asp (RGD) peptides (Peptides International; 0.01 mg/ml) at a volume ratio of 10:1 for 20 min. To conjugate RGD-microbubbles onto the cell surface, the culture medium in the cell culture dish was removed, followed immediately by addition of 50 µl of RGD-microbubble solution. Then the cell culture dish was flipped upside down for 10 min to allow the microbubbles to be attached the cells via RGD-integrin binding. This microbubble concentration was adjusted to achieve a nominal ratio of 1 bubble per 10–20 cells in a hESC colony. The dish was flipped back and gentle washing was performed to remove unbound microbubbles.

### Ultrasound application and single cell sonoporation using targeted microbubbles

As described previously^37,38,40^, sonoporation of hESCs was generated after conjugation of microbubbles to the cells. During experiment, the glass bottom dish with adherent hESCs was placed on the stage of an inverted microscope (Nikon Eclipse Ti-U). A single element planar transducer with central frequency of 1.25 MHz (Advanced Devices, Wakefield, MA, USA; 6 dB beam width of 3.54 mm, Rayleigh distance of 9 mm) was positioned at 45° relative to the horizontal direction, with its active surface submerged in the medium, aiming at the cells. The transducer was driven by a waveform generator (Agilent Technologies 33250A) and a 75 W power amplifier (Amplifier Research 75A250). Before each experiment, a small mental wire was used to align the acoustic field and optical field, and position the transducer 9 mm away from the cells on the dish bottom. A single pulse containing 10 sinusoidal cycles (total duration ~ 8 µs), with peak acoustic pressure of 0.4 MPa, was applied to generate sonoporation of hESCs in this study.

Calibration of the ultrasound transducer was performed in free field using a 40 µm calibrated needle hydrophone (HPM04/1, Precision Acoustics).

Cell viability was determined using Calcein-AM assay (Thermo Fisher) performed after sonoporation.

### Calcium imaging and characterization of intra-and inter-cellular calcium waves

The fluorescent microscopy imaging system used in this study has been described in detail in our previous work^36,46,47^. Briefly, Ca^2+^ indicator fura-2AM was used for monitoring intracellular free Ca^2+^ concentration in hESCs in this study. To load the dye, cells were incubated for 60 min in the incubator in complete cell culture medium containing 10 µM fura-2 AM (Invitrogen, ThermoFisher) and 0.05% v/v of 10% w/v Pluronic F-127 (Invitrogen, Carsbad, CA, USA). After incubation, excess dye was removed by gentle washing. The cell-seeded dish was placed on a 37 °C heating stage on an inverted microscope (Eclipse Ti-U; Nikon, Melville, NY, USA). Real-time fluorescence imaging was performed using a monochromator (DeltaRAM X; PTI, Birmingham, NJ, USA) with 5 nm bandpass to repeatedly filter light from a 75 W xenon lamp at the various wavelengths. The exposure for each channel (340 nm, 380 nm, and 538 nm) was set at 1 ms. The excitation light was directed through a 20 × Super Fluor objective (MRF00200; Nikon, Melville, NY, USA; NA 0.75) to the specimen and the light emitted from the cells was passed through a polychroic filter (73000v2; Chroma, Rockingham, VT, USA) with passbands in the green and red. The resulting series of 16-bit photomicrographs were acquired using a cooled CCD camera (Photometrics Cool Snap HQ, Tucson, AZ, USA) at 512 × 512 resolution. We used Easy Ratio Pro (PTI, Birmingham, NJ, USA) and Image J 1.42 (The National Institutes of Health, Bethesda, MD, USA) for image acquisition and analysis.

The emitted fluorescence intensities at 510 nm from fura-2 in the cells with excitation at two different wavelength (340 nm and 380 nm) were continuously recorded with recording interval of 3.45 ms for two channels, and 5.17 ms for three channels. Total recording time was 6 min (1 min before and 5 min post ultrasound application). The ratio of the emitted intensities from the cells, which is proportional to the intracellular free calcium concentration, was obtained from experimental measurements. Post-processing was performed to generate ratiometric pseudocolor calcium images using a custom Matlab program, where the ratio of background corrected fluorescence intensities of 340 nm to 380 nm was used to encode the hue, while the intensity from 340 nm was used to modulate the display intensity. The intra- and inter-cellular calcium wave speed was quantified from the sequence of ratiometric images recorded during experiments.

### Fluorescent imaging and quantification of cell–cell dye transfer and GJ permeability

Propidium iodide (PI, 668 Da) (Sigma Aldrich) was used as an indicator to visualize the GJIC in hESCs in this study after single cell dye loading using sonoporation^38^. PI is a cell impermeable, nucleic acid intercalating agent, thus only fluoresces (excitation at 538 nm, emission at 610 nm) after entering the cells where nucleic acids are present.

Before experiments for sonoporation, 100 µM PI was added to the culture medium in the cell-seeded dish. Real time fluorescence microscopy was used to record videos of PI fluorescence inside cells after reversible sonoporation, which generated transient membrane disruption allowing loading of PI into single cells followed by subsequent dye transfer into neighboring cells^46^.

#### Estimation of cell–cell permeability using a semi-infinite medium diffusion model

The transient membrane pores generated by sonoporation permitted intracellular uptake of a fixed amount of PI in to single cells targeted by microbubbles. After loading of PI by sonoporation into the sonoporated cell, intracellular diffusion of PI within the sonoporated cell resulted in rapid spread of the molecules in the sonoporated (parent) cell. We assumed that the amount of PI within the parent cell reached a constant after the sonopration pore resealed. The subsequent cell–cell transport occurred through the region of contact between the two cells. Thus transfer of PI from a sonoporated cell to neighboring (recipient or daughter) cells and diffusion in the recipient cells may be effectively modeled as an 1D semi-infinite medium diffusion problem if the observation time is short for a given spatial dimension^48^. Specifically, semi-infinite medium assumption is valid if

1 $$\eta = \frac{x}{{\sqrt {4Dt} }} \ge 3,$$

where *x* is the spatial distance, *D* the diffusion coefficient, and *t* the time. For a typical diffusion coefficient of small molecules 7 × 10^–9^ cm^2^/s and spatial length of 35 µm and observation time of 50 s, $\eta \approx 3$. Therefore under the condition of $\eta > 3$, or *t* > 50 s after sonoporation, we consider PI diffusion from a sonoporated cell (PI concentration was assumed in the sonoporated cell as constant C_1_ due to rapid intracellular diffusion after dye loading) to a neighboring recipient cell with PI concentration of C_2_(x, t) as a 1D semi-infinite medium diffusion problem with the following equation of diffusion and initial condition,

2 $$\frac{{\partial C_{2} }}{\partial t} = D\frac{{\partial^{2} C_{2} }}{{\partial x^{2} }},$$

3 $$C_{2} = 0, \; x > 0, \;t = 0.$$

In addition, the rate of PI transport at the interface of the two cells ($x = 0$) is proportional to the concentration difference between the sonoporated cell and recipient cell,

4 $$- D\frac{{\partial C_{2} }}{\partial x} = k\left( {C_{1} - C_{2} } \right), \;x = 0,$$

where $k$ is the permeability of the cell–cell barrier, which is the GJ permeability for molecular exchange between the adjacent cells.

We perform Laplace transform on Eq. (2) and considering the initial condition in Eq. (3), we obtain

5 $$D\frac{{\partial^{2} \overline{{C_{2} }} }}{{\partial x^{2} }} = p\overline{{C_{2} }} ,$$

where $\overline{{C_{2} }}$ is the Laplace transform of $C_{2} \left( {x,t} \right)$. The boundary condition, Eq. (3), becomes

6 $$- D\frac{{\partial \overline{{C_{2} }} }}{\partial x} = \frac{{kC_{1} }}{p} - k\overline{{C_{2} }} , x = 0.$$

Solving Eq. (5) while considering Eq. (6), we obtain

7 $$\frac{{\overline{{C_{2} }} }}{{C_{1} }} = \frac{h}{{p\left( {q + h} \right)}}e^{ - qx} ,$$

where $h = k/D$ and $q = \sqrt {p/D}$. Performing inverse Laplace transform, we obtain the PI concentration in the recipient cell as a function of time and location^49^

8 $$\frac{{C_{2} \left( {x,t} \right)}}{{C_{1} }} = erfc\left( {\frac{x}{{2\sqrt {Dt} }}} \right) - \exp \left( {\frac{k}{D}x + \frac{{k^{2} t}}{D}} \right) \times erfc\left( {\frac{x}{{2\sqrt {Dt} }} + k\sqrt {\frac{t}{D}} } \right),$$

Equation (8) is then used to estimate cell–cell permeability (or GJ permeability) $k$ and diffusion coefficient $D$ based on experimentally measured PI fluorescence intensity in a recipient cell. We determined a straight line inside the recipient cell perpendicular to the GJ plane to indicate spatial locations from the cell barrier. Along this line, PI fluorescence intensity values were extracted from recorded images at different time point, and fit to Eq. (8). Since the cell nucleus has high concentration of nucleic acids, which results in much higher PI fluorescence intensity in the nucleus than that in the cytosol, we excluded the nuclear PI data in model fitting and only used the PI data in the cytosol.

#### Estimation of cell–cell permeability using a quasi-steady state diffusion model

We also use a quasi-steady state diffusion model in this study for estimation of cell–cell permeability. In this model, we consider the average concentration of PI in a cell as a function of time without considering spatial variation, thus making the model a lumped parameter or compartmental model. We also regard the GJ as a thin, plane barrier separating two cells. Due to the small scale of the thin barrier compared to the volume of the cells, changes in PI concentration in a sonoporated cell, C_1_(t), and in a recipient cell, C_2_(t), are much slower than diffusion across the thin GJ plane. Thus molecule diffusion through the thin GJ barrier from a sonoporated cell to a neighboring recipient cell can be considered as a quasi-steady-state diffusion problem with the boundary conditions being the constant PI concentration in the two adjacent cells^48^. The diffusion equation within the thin barrier is thus

9 $$\frac{{\partial C_{m} }}{\partial t} \approx 0 \approx D_{m} \frac{{d^{2} C_{m} }}{{dy^{2} }},$$

where *D*_*m*_ is the diffusion coefficient of PI within the GJ barrier, and *y* is the spatial location within the barrier. Equation (9) has a solution

10 $$C_{m} \left( y \right) = \Phi C_{1} - \Phi \left( {C_{1} - C_{2} } \right)\frac{y}{L} ,$$

where Φ is the partition coefficient, $L$ the thickness of the GJ barrier, *y* the spatial location within the membrane. The flux of PI across the barrier is obtained as

11 $$J = - D_{m} \frac{{dC_{m} }}{dx} = \frac{{D_{m} \Phi }}{L}\left( {C_{1} - C_{2} } \right) = k\left( {C_{1} - C_{2} } \right),$$

where $k = \frac{{D_{m} \Phi }}{L}$ is the permeability of the GJ barrier between two cells.

To find the concentration in the recipient cell $C_{2}$, we consider mass balance in the cell.

$$\left[ {Volume \times rate\; of\; increase\; of\;PI\; in\;cell\;2} \right] = \left[ {Area \times influx\;of\;PI\;into\;cell2\;across\;the\;GJ\;from\;cell\;1} \right]$$

which can be expressed mathematically as,

12 $$V_{2} \frac{{dC_{2} }}{dt} = JA_{m} = kA_{m} \left( {C_{1} - C_{2} } \right),$$

where $V_{2}$ is the volume of recipient cell 2, $A_{m}$ is the area of GJ through which cell–cell transport occurs between the two cells.

Since a fixed amount of PI was loaded into a cell by sonoporation, concentration in the sonoporated cell, $C_{1}$, can be regarded as constant after the initial increase. Generally, $C_{1} \gg C_{2}$ and $C_{2} \left( {t = 0} \right) = 0,$ thus solution for Eq. (12) is obtained

13 $$\ln \left( {C_{1} - C_{2} } \right) = - \frac{{kA_{m} }}{{V_{2} }}t + \ln C_{1} ,$$

or,

14 $$\frac{{C_{2} \left( t \right)}}{{C_{1} }} = 1 - \exp \left( { - \frac{{kA_{m} }}{{V_{2} }}t} \right).$$

We use the PI fluorescence intensity from cell 1 (donor cell) and cell 2 (recipient cell) extracted from experimental recordings to fit Eq. (14) to estimate GJ permeability $k$. To meet the condition for the model, only data after a time period when $C_{1}$ reaches constant were used in model fitting. Cell volume was estimated by the product of measured cell area (from images) and a height of 5 µm. The area representing functional GJ was estimated from lateral length of connection between cells from images and a cell height of 5 µm.

## Results

### Sonoporation enabled single cell dye loading and dynamic visualization of GJIC in hESCs

**Figure 1:**
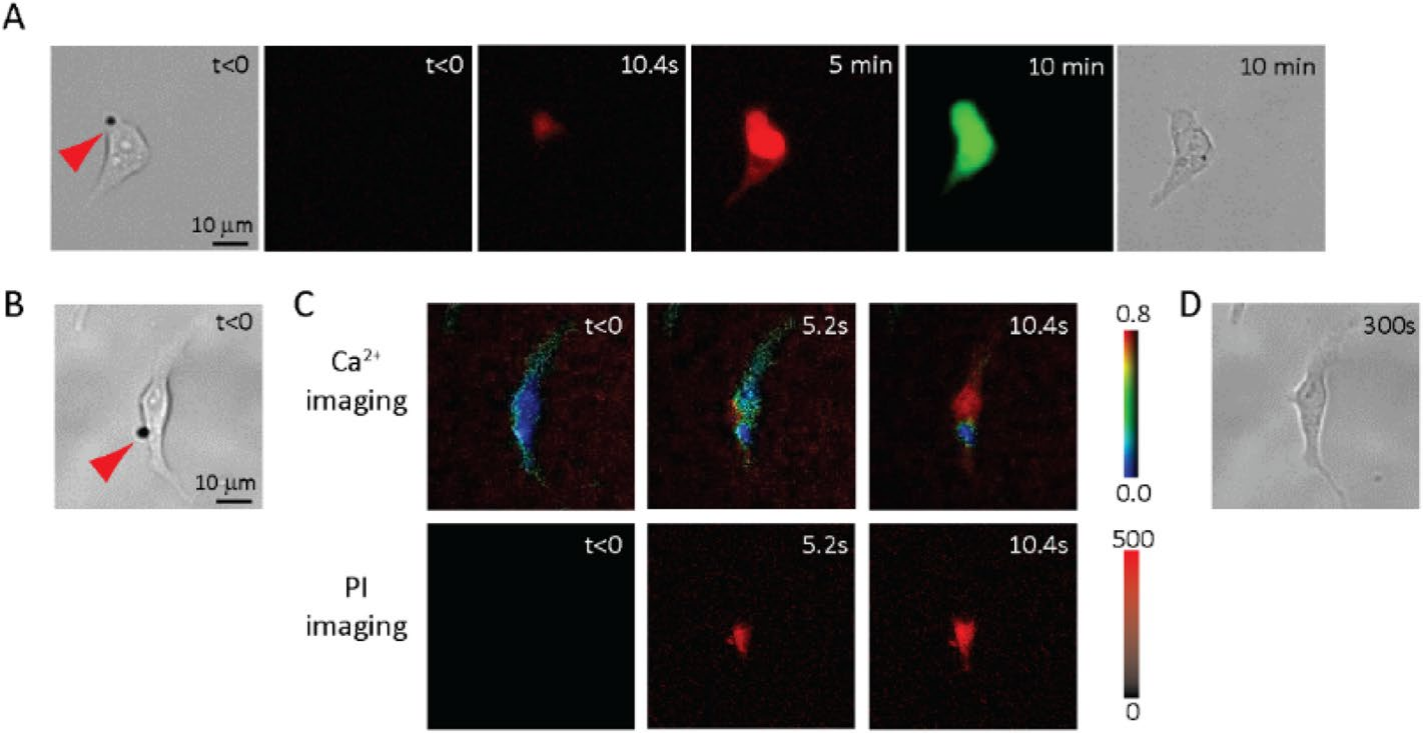
Microbubble mediated sonoporation allows intracellular uptake of propidium iodide (PI) and influx of Ca^2+^ in human embryonic stem cells (hESCs). (**A**) Sonoporation of a single hESC with an attached microbubble (arrow in the first bright field image before application of ultrasound pulse t < 0) resulted in intracellular uptake of PI (red fluorescent signal) into the cell from the site of sonoporation. Calcein staining (green) confirmed cell viability after sonoporation (t = 10 min). (**B**) A hESC with an attached microbubble before sonoporation. (**C**) Simultaneous fluorescence imaging of intracellular Ca^2+^ and PI showing molecular entry after sonoporation. (**D**) The cell in (**B**) after sonoporation. Ultrasound pulse duration 8 µs, acoustic pressure 0.4 MPa.

Microbubbles functionalized with RGD were first stably attached to the surface of adherent hESCs via RGD-integrin binding (Fig. 1A,B). A short ultrasound pulse (duration 8 µs, acoustic pressure 0.4 MPa) was applied to induce single cell sonoporation^38^ by acoustic cavitation of the attached microbubbles (radius 1–2 µm) (Fig. 1A,B). Sonoporation generated transient pores on the cell membrane^38,41,43^, resulting in intracellular uptake of propidium iodide (PI) molecules without affecting cell viability, as assessed by calcein-AM assay (Thermo Fisher) performed 10 min after sonoporation (Fig. 1A), similar to what we reported before due to a transient (lasting for ~ 5 s), small (5–20 nm) pore on the cell membrane^38,43^. As in other cell types^36,38,46,50^, sonoporation by an attached microbubble (Fig. 1B) also generated an influx of extracellular Ca^2+^ in hESCs (Fig. 1C,D), indicating that these phenomena are independent of cell types.

**Figure 2:**
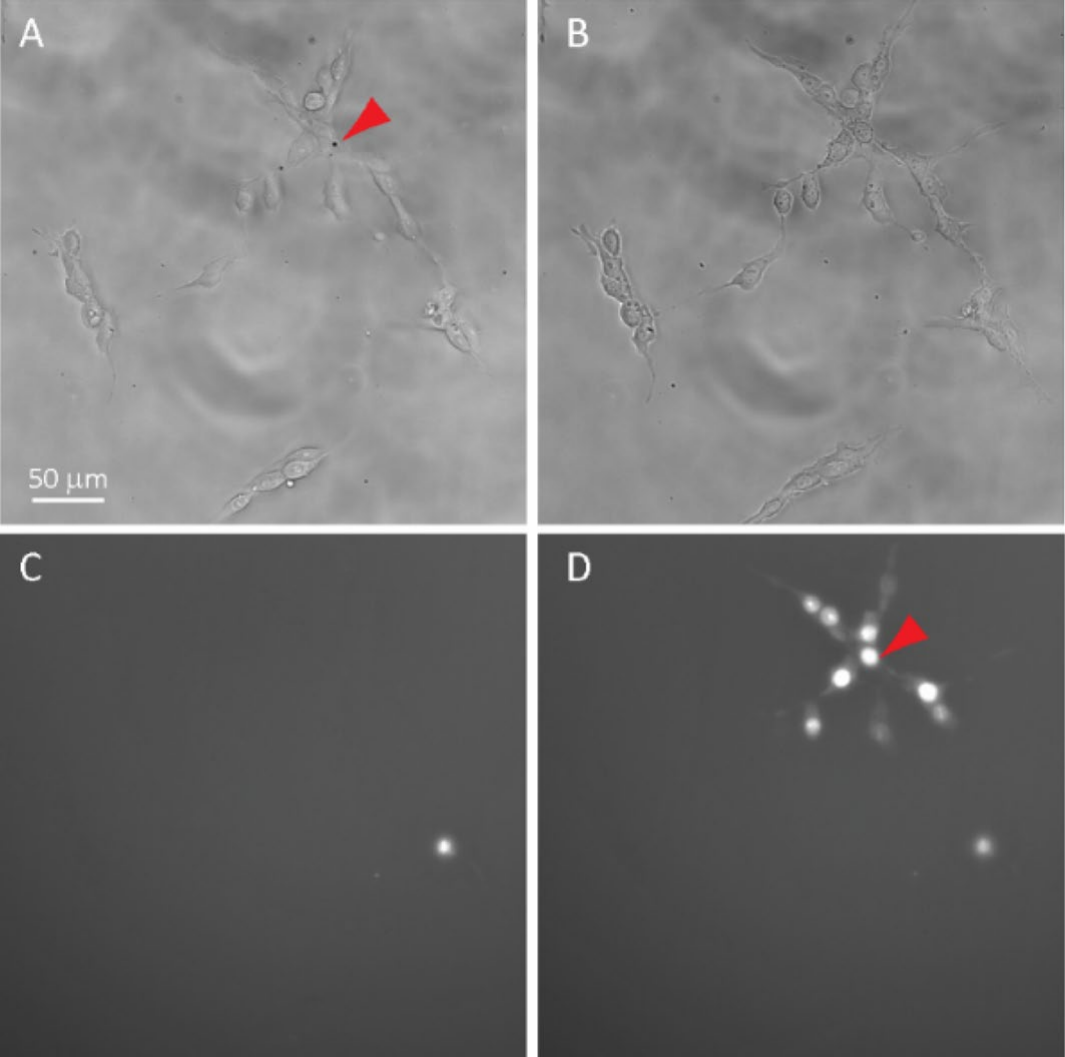
Single cell loading of propidium iodide (PI) by sonoporation and cell–cell dye transfer via gap junctions (GJs) in hESCs. (**A**) Bright field image of hESCs before sonoporation with a cell-attached microbubble (arrow). (**B**) Bright field image of hESCs after sonoporation. (**C**) PI fluorescent image before sonoporation. (**D**) PI fluorescent image of hESCs after sonoporation at steady state, showing the uptake of PI in the sonoporated cell (arrow) and the surrounding cells after cell–cell dye transfer via GJs.

**Figure 3:**
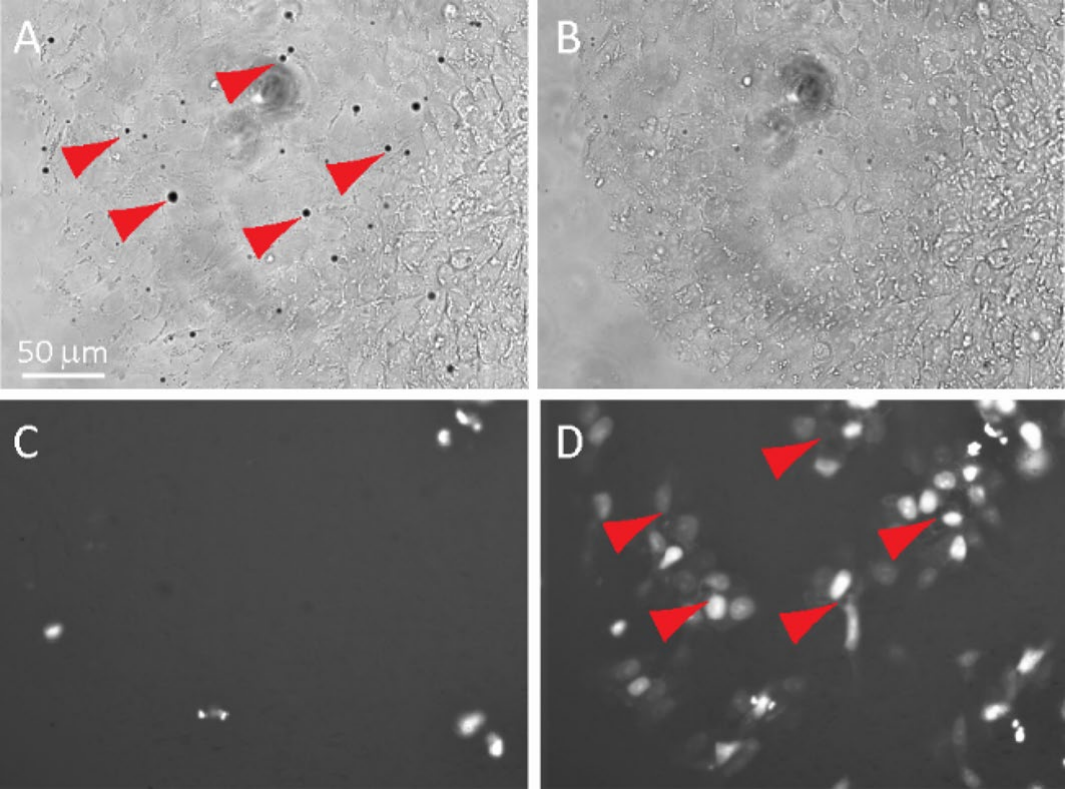
Dye (PI) loading into multiple single cells by sonoporation and cell–cell dye transfer via gap junctions (GJs) in a hESC colony. (**A**) Bright field image before sonoporation with microbubbles (arrows) attached to multiple cells. (**B**) Bright field image of hESCs after sonoporation. (**C**) Fluorescent image of PI before sonoporation. Dead cells show high PI intensity that were not related to sonoporation. (**D**) Fluorescent image showing intracellular uptake of PI in the cells with attached microbubbles in (**A**) (arrow) after sonoporation and surrounding cells due to subsequent cell–cell dye transfer via GJs.

Dye transfer to neighboring cells after sonoporation-induced PI loading clearly revealed the functional GJIC in the cells (Fig. 2, Movie S1). For imaging GJIC in hESCs in a colony (Fig. 3), sonoporation was applied to enable rapid PI loading into multiple cells simultaneously (Fig. 3A), followed by dye transfer to neighboring cells (Movie S2, Fig. 3D).

### Determination of cell–cell permeability between hESCs

**Figure 4:**
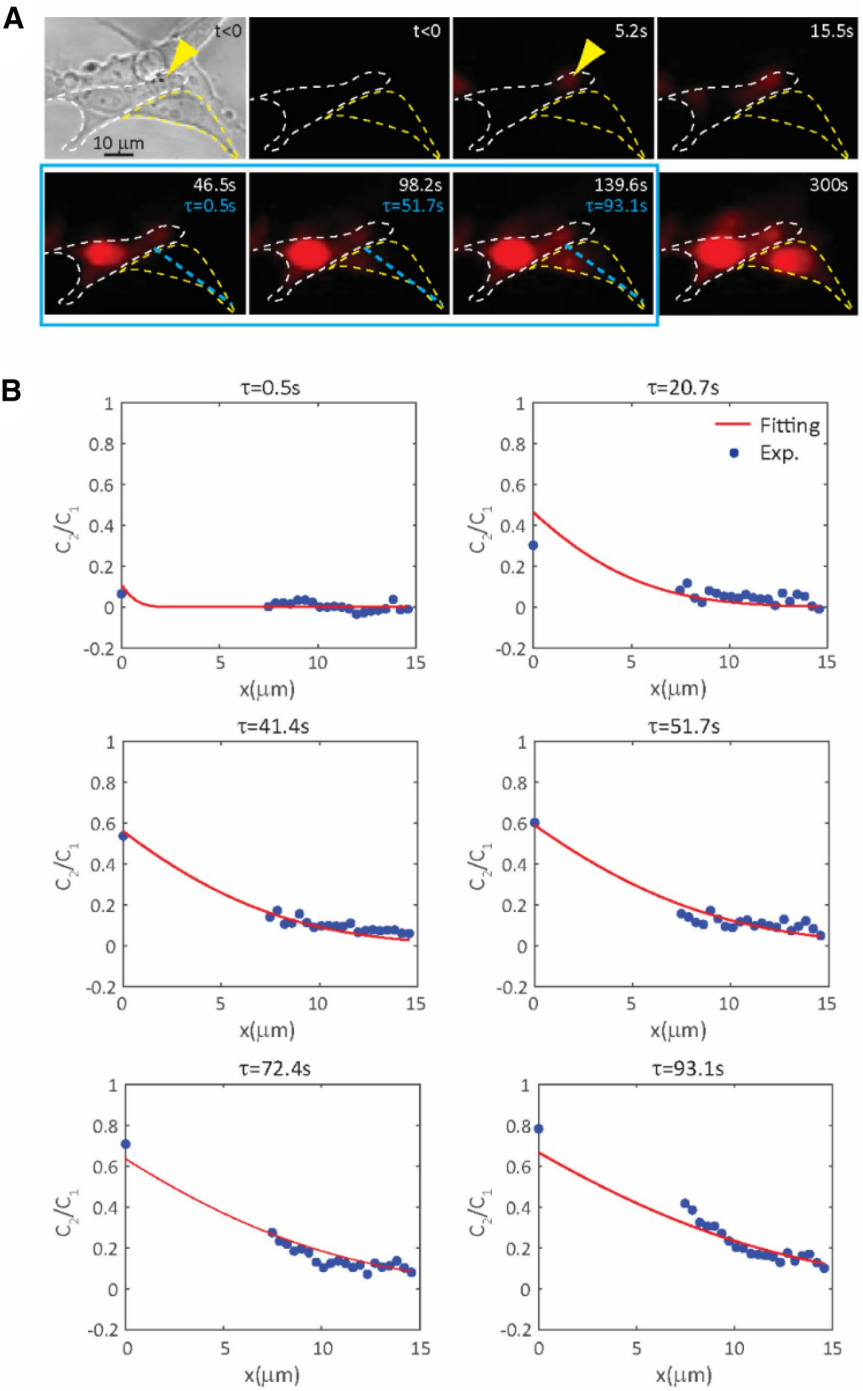
Determination of gap junction (GJ) permeability from cell–cell propidium iodide (PI) transfer and subsequent intracellular diffusion using an 1D semi-infinite medium diffusion model. (**A**) Sonoporation (*t* = 0) of a single cell (white dashed outline) via a membrane-bound microbubble (yellow arrow) permitted intracellular uptake of PI and PI transport into a neighboring cell (yellow dashed outline). Data from the blue highlighted frames represent steady state of PI in the sonoporated cell and were used for model fitting. Blue dashed line within the recipient cell indicates the 1D spatial distance from the GJ separating the sonoporated cell and recipient cell. (**B**) Plots of normalized PI concentration from model prediction and experimental data along the blue dash line within the recipient cell in (A) at different time points. Data points representing PI intensity within the nucleus of the recipient cell were not shown and not used in model fitting. Ultrasound pulse duration was 8 µs, acoustic pressure 0.4 MPa.

Formation of a small (e.g. 10–30 nm) and transient pore (2–5 s) in sonoporation^38,41,43^ enabled a fixed amount of extracellular PI or Ca^2+^ to enter the cells. We assessed GJIC based on subsequent diffusion of these molecules to neighboring cells (Fig. 4A, Movie S3).

Under the condition described in Materials and Methods, we applied a 1D semi-infinite diffusion model to study the transport of PI from a sonoporated cell to an adjacent cell (Fig. 4A). Due to relatively faster processes of PI-nucleic acids binding and intracellular diffusion of PI molecules within the sonoporated cell compared to transport across GJs, we considered PI concentration in a sonoporated cell approximately constant for Eq. (8), which was confirmed in experiments that after a period of time after dye loading, the PI fluorescence intensity plateaued. Therefore we only used data after this time period for model fitting. For example, only data after 46.5 s in Fig. 4 were used.

PI fluorescence intensity in the nucleus is much stronger than that in the cytoplasm because of higher nucleic acids concentration in the nucleus. To avoid making assumptions of nucleic acid concentrations, we only used PI data in the cytoplasm for model fitting of Eq. (8) (Fig. 4B) and obtained GJ permeability *k* = (0.156 ± 0.033) µm/s (*n* = 9) and diffusion coefficient *D* = (7.40 ± 3.3) × 10^–9^ cm^2^/s (*n* = 9), which are consistent with reported values in other cells. For example, the previously reported unitary permeability of GJ channels to second messengers cAMP and InsP_3_ as well as LY for Hela cells^51^, when converted to cell–cell permeability, was 0.62 µm/s, 0.79 µm/s, and 0.092 µm/s, respectively, assuming a cell–cell contact area of 25 µm^2^ and a number of 330 GJ channels^51^. Our results are also comparable with the GJ permeability reported for calcein^52^, 0.088 to 4.2 µm/s, after conversion to cell–cell permeability, assuming a cell–cell contact area of 25 µm^2^ and 110 GJ channels^52^.

### Measurement of cell–cell permeability in hESC colonies

As described in Materials and Methods, we also employed a lumped-parameter model of dye coupling between sonoporated cells and recipient cells to estimate GJ permeability. In this model, the total PI signal in a cell was considered without spatial dependence, and change of PI intensity vs. time was utilized to obtain cell–cell permeability.

**Figure 5:**
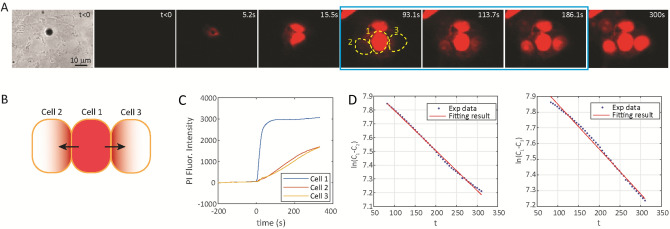
Estimation of gap junction (GJ) permeability using a quasi-steady state diffusion model. (**A**) Sequence of images show that microbubble mediated sonoporation induced influx of propidium iodide (PI) in a single cell (cell 1), followed by PI diffusion into two neighboring cells (cell 2 and cell 3). The three images within the blue frame were used for model fitting. Ultrasound pulse (duration 8 µs, 0.4 MPa) was applied at *t* = 0. (**B**) Schematic illustration of quasi-steady state diffusion from sonoporated cell (cell 1) to neighboring cells. (**C**) Measured PI fluorescence intensity over time in cell 1, 2, and 3 in (**A**). (**D**) Model fitting of measured PI intensities for GJ permeability estimation using data from cell 1, 2, and 3 in (**A**).

As shown in Fig. 5 and illustrated by Eq. (14), we examined PI coupling after sonoporation (Fig. 5B, Movie S4) based on the total intracellular PI intensity in the whole cell over time. Satisfying the assumption for the diffusion model, we only used PI data after the intensity reached constant in the sonoporated cell (from 93.1–186.1 s, Fig. 5A,C) for fitting Eq. (14) (Fig. 5D), and obtained cell–cell permeability *k* = (0.139 ± 0.038) µm/s (*n* = 20), a value comparable with the result obtained using the 1D semi-infinite medium diffusion model described in the previous section. Here we used a cell volume (as the product of cell area and a height of 5 µm) of 1150 µm^3^ and functional GJ area of 25 µm^2^ for our model.

### Heterogeneous distribution of GJIC in hESCs

**Figure 6:**
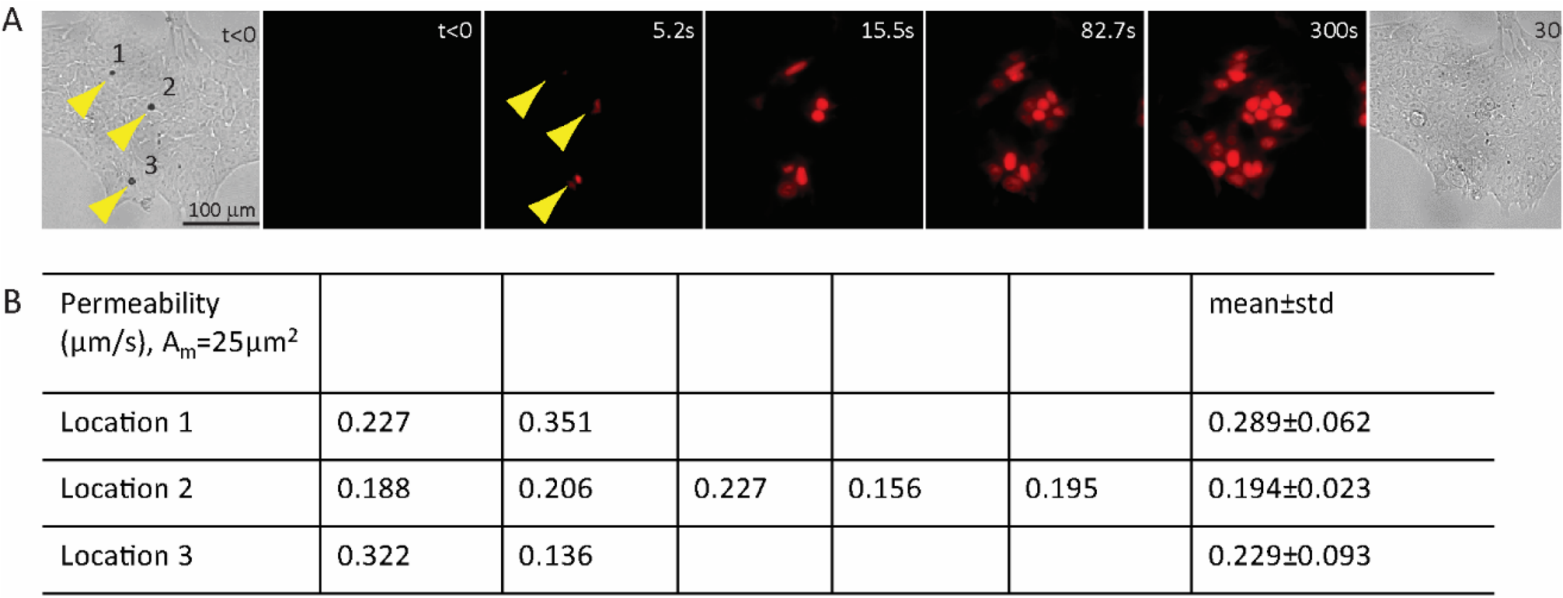
Estimation of gap junction (GJ) permeability in multiple cells using a quasi-steady state diffusion model. (**A**) Sonoporation of multiple single cells facilitated by attached microbubbles (arrows in the first bright field image) in a hESC colony for PI loading, followed by dye transfer through GJs into neighboring cells. (**B**) Calculated values of GJ permeability for different GJs at the three locations marked in (**A**). The area of GJ was assume to be 25 µm^2^.

Using the above lumped-parameter, compartment model of mass transport, we obtained the cell–cell permeability or the permeability of functional GJs of hESCs at multiple locations in the same colony (Fig. 6, Movie S5). Notably in these cases, a varying number of dye coupling events were detected surrounding different sonoporated cells (Fig. 6A, Movie S5), although the sonoporated (donor) cells were surrounded by other cells in a similar fashion in the colony. For example, PI transfers from a sonoporated cell to two adjacent cells (Fig. 6A) were detected at location 1, whereas PI transfer from a sonoporated donor cell to five adjacent recipient cells were detected at location 2, suggesting inhomogeneous distributions of functional GJs in hESCs, although the average GJ permeability values for the functional GJs between different pair of cells (Fig. 6B) were similar at different locations.

We conducted further experiments to assess the extent and progression of functional cell–cell communications in hESCs. Our results show that after 1 day of culture, there were 17.2 ± 6.89% (*n* = 3) of cells adjacent to sonoporated cells that exhibited detectable PI coupling from sonoporated cells (total of 39 sonoporated cells), suggesting the establishment of cell–cell communications in hESC colonies after 1 day of culture. The percentage of neighboring cells with PI coupling from a sonoporated cell increased after 4 day and 8 day of culture, to 30.8 ± 9.57% (*n* = 3, total 41 sonoporated cells ) on day 4 and 32.6 ± 6.42% (*n* = 3; total 35 sonoporated cells) on day 8, respectively. However, the increases of PI coupling between cells in close range were not statistically significant.

We found that culture duration did not affect the permeability values of functional GJs surrounding a sonoporated cell (Fig. S1A). However, the spatial range of PI diffusion from sonoporated cells in terms of the number of cells exhibiting PI uptake increased with culture time (Fig. S1B). Specifically, the average number of successive cells with PI uptake associated with a sonoporated cell was 5.1 ± 2.5 cells (*n* = 35) on day 1 and increased to 6.14 ± 3.1 cells (*n* = 25) on day 4 and 8.4 ± 4.0 cells (*n* = 18) on day 8, suggesting increased longer range cell–cell connectivity in a hESC colony over time.

Immunostaining of Connexin 43 in hESCs confirmed the protein expression in the cells, but did not show conclusive differences in expression pattern (Fig. S2) to correlate with the functional hererogeneity in GJIC observed in this study.

### Sonoporation of single hESCs induced Ca^2+^ waves

With higher extracellular Ca^2+^ concentration than [Ca^2+^]_i_, influx of Ca^2+^ into the intracellular cytoplasm effectively increased [Ca^2+^]_i_ in the sonoporated cells (Fig. 1C). Interestingly, increases of [Ca^2+^]_i_ in other non-sonoporated hESCs in a colony were also observed after sonoporation (Fig. S2, Movie S6). The spatiotemporal changes of [Ca^2+^]_i_ in non-sonoporated hESCs exhibited a wave-like behavior, as intercellular calcium waves. Diffusion of intracellular Ca^2+^ through GJs likely played a role in the generation of intercellular calcium waves initiated from sonoporated cells.

**Figure 7:**
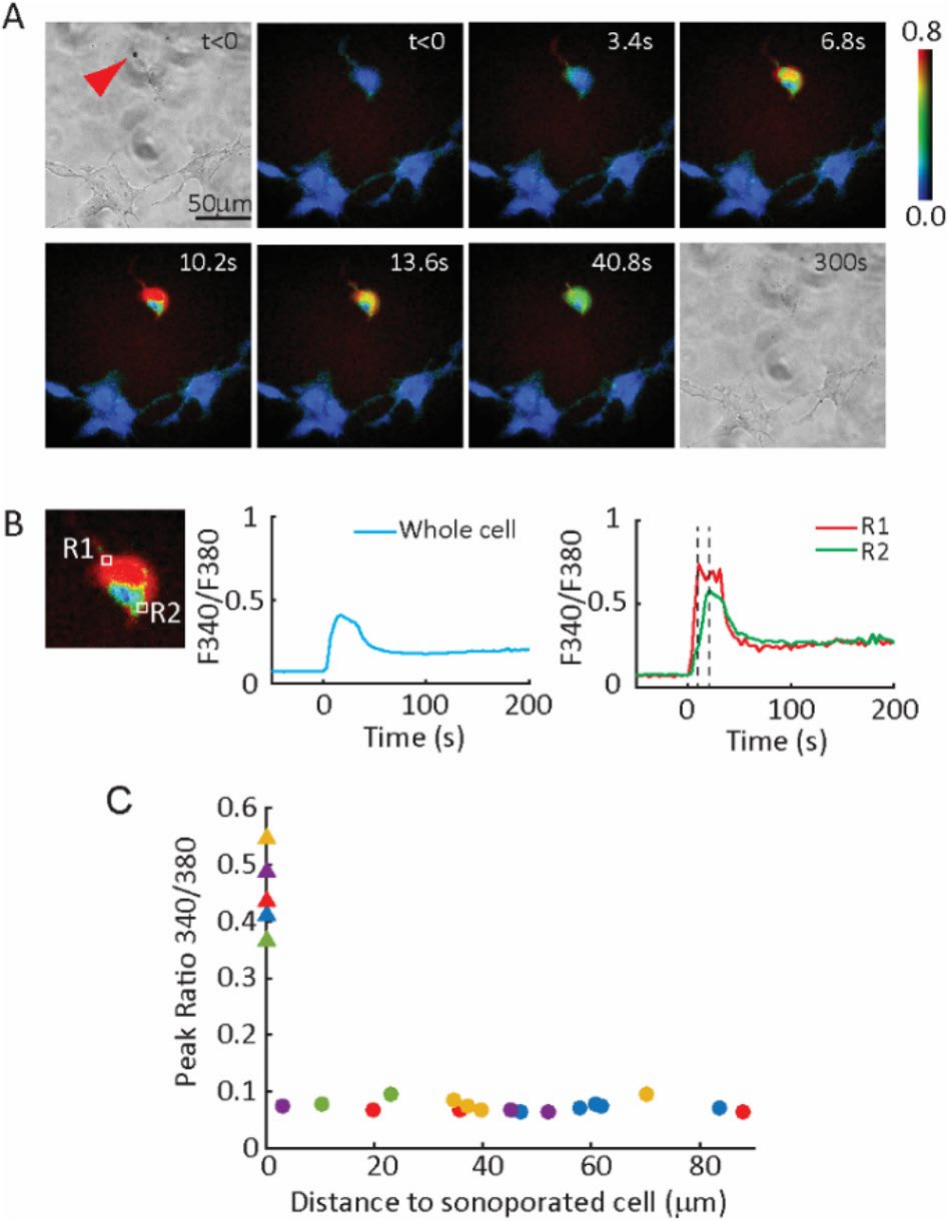
Intercellular Ca^2+^ waves in hESCs required cell–cell contact. (**A**) Sequence of images showing that cavitation of a microbubble (arrow) by a short ultrasound pulse (8 µs, 0.4 MPa) induced Ca^2+^ influx into the cell. (**B**) Zoomed-in image of the sonoporated hESC in (**A**) with selected intracellular regions (R1 and R2). The plots show Fura2 fluorescence ratio (340 nm/380 nm) over time for the whole cell, as well as R1 and R2. Time delay between R1 and R2 represented intracellular Ca^2+^ diffusion within the sonoporated cell. (**C**) Scatter plot of experimental data showing peak Fura2 fluorescence ratio (340 nm/380 nm) in sonoporated hESCs (colored triangles) and the corresponding neighboring hESCs (colored circles) located at different distances. Triangle and circles with the same color indicate measurements from the same field of view in one experiment.

To verify this, we conducted experiments using isolated hESCs without cell–cell contacts. Here sonoporation generated influx of Ca^2+^ into a single cell (Fig. 7A, Movie S7). Intracellular diffusion of Ca^2+^ within the cytosol, which sometimes is described as intracellular Ca^2+^ wave phenomelogically, resulted in a speed of 5.08 ± 0.34 µm/s (*n* = 6) estimated from the increase of fluorescent signals within the cells (Fig. 7B). This value is consistent with the time scale of passive diffusion assuming a diffusion coefficient of Ca^2+^ in the cytosol^53^ at 5.3 × 10^–6^ cm^2^/s.

No changes in [Ca^2+^]_i_ were detected in other hESCs that were not in direct contact with the sonoporated cell (Fig. 7A,C, Movie S7), even when the cells were nearby (Fig. S3, Movie S8), suggesting that direct cell–cell contact and communication through GJs were required in intercellular Ca^2+^ waves in hESCs observed in this study.

### Characteristics of intercellular Ca^2+^ waves and Ca^2+^ signaling in hESCs

Increases of [Ca^2+^]_i_ in hESCs can also come from other sources. For example, Ca^2+^-induced Ca^2+^ release from internal stores and calcium signaling^54^ can result in increase of [Ca^2+^]_i_ patterns different from simple diffusion patterns. Indeed, we observed a variety of intercellular calcium wave patterns in this study.

**Figure 8:**
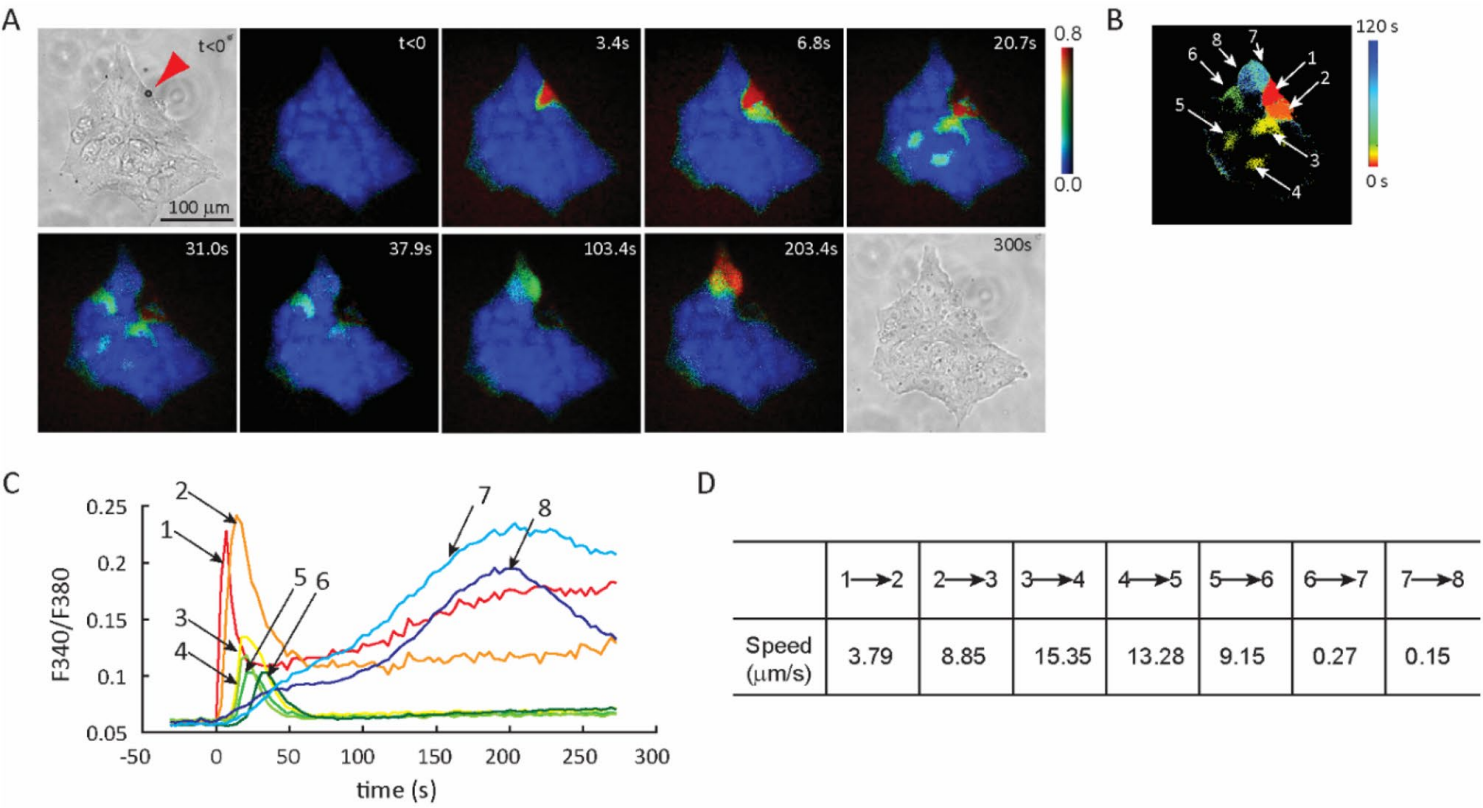
Single cell sonoporation enabled visualization of intercellular calcium wave in a hESC colony with high spatiotemporal resolution. (**A**) Sequential images show that facilitated by a microbubble (red arrow), a single ultrasound pulse (8 µs, 0.4 MPa) induced Ca^2+^ influx in sonoporated cell (cell 1), initiating intercellular Ca^2+^ wave that propagated to cell 2 to 8 in a discontinuous fashion. (**B**) Heat map showing the time sequence of intracellular Ca^2+^ concentration increase in cell 1–8, indicating the Ca^2+^ wave propagated in a circle. (**C**) Plot of Fura2 fluorescence ratio (340 nm/380 nm) showing the delay of increase in Ca^2+^ concentration for cell 1–8. (**D**) Cell–cell Ca^2+^ wave speed.

In the example shown in Fig. 8 and Movie S9, intercellular Ca^2+^ wave exhibited complex spatiotemporal pattern. As shown in experiments, dye loading and initial increase of [Ca^2+^]_i_ only occurred in cells with attached microbubbles, no Ca^2+^ waves were generated in experiments with ultrasound but without microbubbles or with microbubbles but without ultrasound. Besides rapid increase of [Ca^2+^]_i_ in a sonoporated cell (cell 1) (Fig. 8A), spatial discontinuity and non-linear path were observed in the Ca^2+^ wave. The temporal change of [Ca^2+^]_i_ in hESCs did not correlate with their spatial locations relative to the sonoporated cell (cell 1). Cell 7 and cell 8 were situated closer to the sonoporated cell (cell 1) than cell 4 and cell 5; however, they exhibited an increase of [Ca^2+^]_i_ later than cell 4 and cell 5 (Fig. 8B,C). It is also clear from the image that not all hESCs surrounding the sonoporated cell (cell 1) exhibited changes in [Ca^2+^]_i_ following the increase of [Ca^2+^]_i_ in cell 1 (Fig. 8B). The observed discontinued Ca^2+^ wave pattern cannot be explained by diffusion of extracellular signaling molecules, as such diffusion would likely not be restricted to discrete cells within the colony. However, Ca^2+^ wave can be triggered by intercellular diffusion of other signaling molecules that were not imaged and the complex Ca^2+^ wave propagation pattern eflects the heterogeneous cell–cell connection network.

In addition, [Ca^2+^]_i_ exhibited different characteristics in both initiation time and temporal profile in different cells (Fig. 8C). Furthermore, the speed of calcium wave exhibited different values from cell to cell (Fig. 8D), suggesting variation in GJ permeability in the colony. Besides calcium signaling in hESCs, these complex [Ca^2+^]_i_ wave patterns may also be indicative of non-uniform distribution of functional GJs, made visible by Ca^2+^ activities initiated from single cells.

**Figure 9:**
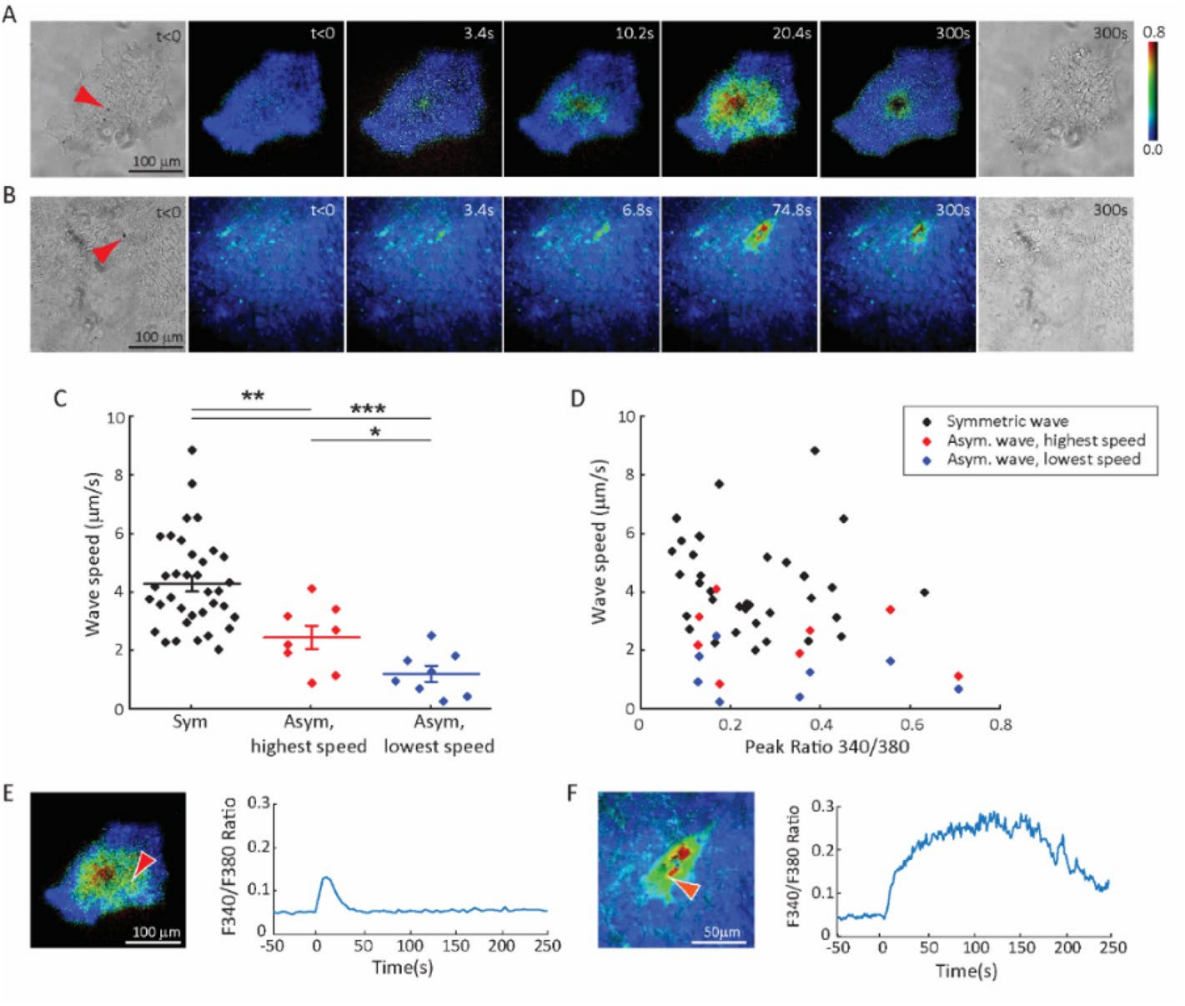
Symmetric and asymmetric intercellular Ca^2+^ waves in hESCs induced by single cell sonoporation. (**A**) A symmetric Ca^2+^ wave initiated from a single cell by sonoporation via a microbubble (arrow). (**B**) An asymmetric Ca^2+^ wave initiated from a single cell by sonoporation via a microbubble (arrow). (**C**) Wave speed for symmetric waves (4.31 ± 1.55 µm/s; *n* = 36), as well as the fastest speed (2.56 ± 1.03 µm/s; *n* = 8) and slowest speed (1.22 ± 0.72 µm/s; *n* = 8) for asymmetric waves. (**D**) Scatter plot of wave speed and peak Fura2 fluorescence ratio (340 nm/380 nm) in sonoporated cells. (**E**) Fura2 fluorescence intensity ratio in a single cell (arrow) in (**A**) after sonoporation showing increase and recovery of intracellular Ca^2+^ concentration. (**F**) Fura2 fluorescence intensity ratio in a cell (arrow) in (**B**) showing increase and decrease as well as Ca^2+^ concentration oscillation (at 170—200 s) after single cell sonoporation. Ultrasound pulse duration was 8 µs with an acoustic pressure of 0.4 MPa.

Both radially symmetric (Fig. 9A, Movie S10) and asymmetric Ca^2+^ waves (Fig. 9B, Movie S11) were observed. Symmetric calcium waves exhibited a wave speed (4.31 ± 1.55 µm/s; *n* = 36) greater than asymmetric waves (2.56 ± 1.03 µm/s in the fastest direction and 2.01 ± 0.72 µm/s in the orthogonal direction; *n* = 8) (Fig. 9C). While a range of calcium wave speed values were detected for hESCs, calcium wave speed was not correlated with the values of [Ca^2+^]_i_ in sonoporated cells (Fig. 9D), suggesting that calcium wave speed was an intrinsic property of hESCs independent of the amount of Ca^2+^ influx due to sonoporation. The temporal characteristics of [Ca^2+^]_i_ were also different for cells in symmetric and asymmetric waves (Fig. 9E,F). Calcium oscillation was also observed (Fig. 9F, Movie S11), again indicating calcium signaling beyond simple Ca^2+^ diffusion.

## Discussion

Direct molecular transport between cells through GJs has been well-documented as the mechanism for molecular exchange between adjacent cells. Recently, tunneling nanotubes (TNTs) have been discovered as a new route of direct cell‐to‐cell communication^55–57^. TNTs, formed as thin membrane channels between mammalian cells^55–57^, have been observed to facilitate long‐range communication between dislodged cells. Found in a number of cell types and particularly in infected cells, there existence in hESCs are unknown. In this study, our results clearly show the robust cell–cell transport occurring exclusively in the cells that were in contact with the sonoporated cells. Therefore in this study, we regard the observed cell–cell molecular coupling as the result of transport through GJs in hESCs.

Our results demonstrate the feasibility of using sonoporation as a unique and advantageous strategy for dye loading into single live cells, compared to studies on GJ transport using conventional methods including scrape loading and microinjection. Our technique enables rapid dye loading into multiple single cells simultaneously, allowing assessment of GJ permeability at multiple sites with higher throughput, compared to single cell technique such as microinjection, and with single cell resolution, compared to techniques such as scrape loading.

We assumed the molecular transport from the sonoporated cell to an adjacent cell and diffusion as a 1D model. Considering the substantial contact area between the connected cells, this model is an approximation under the conditions described in the method section if the diffusion within a time limit is considered. Under this assumption, a close form solution is mathematically obtained so that experimental data are fitted to the model to obtain cell–cell permeability. Theoretically, the diffusion problem can be solved based on the mass transport equation with actual cell shapes and dimension without the 1D assumption. However, general treatment like this can only yield numerical solutions which do not provide the benefit of explicit relations of permeability in terms of the spatiotemporal dye concentration distribution.

Our results show heterogeneous intercellular connectivity and Ca^2+^ wave characteristics in hESC colonies. Further studies are needed to examine the implication of these findings. For example, connexin expression in human pluripotent stem cells (hPSCs) has been found to be dramatically different between the pluripotent “naïve state” and the “primed state”^58^. Sonoporation may provide a tool to determine whether the changes in GJIC can serve as a functional biomarker for the pluripotency continuum in stem cells. The technique can also be used for investigating GJIC and Ca^2+^ in hPSCs to determine alteration of GJIC as the result of pathology or pharmacological and genetic intervention.

Similar to conventional studies of GJIC, we used fluorescent tracers to visualize molecule transport between cells. The use of PI enabled uninterrupted, continuous imaging of dynamic intercellular dye coupling without the need to wash after dye loading. Our estimation of GJ permeability relies on assumptions of cell volumes and areas separating two adjacent cells from microscopic images, although these factors do not impact assessment of overall cell–cell communication. Sonoporation should also work for other dye molecules, and it may be worth to further examine whether different dye molecules resulted in different GJ transport patterns.

Unlike conventional methods that use chemical agents to invoke calcium activities in a population of cells without spatial distinction, microbubble-facilitated sonoporation initiates Ca^2+^ activities from single hESCs, a capability particularly useful for investigating GJIC networks and Ca^2+^ signaling with high spatiotemporal resolution. As a universal carrier of biological signals^54^, Ca^2+^ controls numerous cell functions, including cell proliferation and apoptosis^59^. Ca^2+^ signaling is critical for proliferation and directed differentiation of hESCs^60,61^, although details of Ca^2+^ signaling and regulation of intracellular Ca^2+^ concentration ([Ca^2+^]_i_) in hESCs are incompletely understood^44,45^. Information of the amplitude, range, and heterogeneity of GJIC in hESCs could be useful for experimental investigation and mathematical modeling of hESC behaviors.

Although this study used hESCs to demonstrate the feasibility of sonoporation for studying GJIC and Ca^2+^ signaling, our technique is readily applicable to other cell types for quantifying GJIC as a functional biomarker for assessing disease progression, adverse effects of toxicants^4^, chemical carcinogenesis^62^, and efficacy of drugs^29^.

## Supplementary information

Supplementary Movie 1.

Supplementary Movie 2.

Supplementary Movie 3.

Supplementary Movie 4.

Supplementary Movie 5.

Supplementary Movie 6.

Supplementary Movie 7.

Supplementary Movie 8.

Supplementary Movie 9.

Supplementary Movie 10.

Supplementary Movie 11.

Supplementary Information.
