# Supplementary material for: Visualization and quantification of dynamic intercellular coupling in human embryonic stem cells using single cell sonoporation
Source: Sci Rep. 2020 Oct 26;10:18253. doi: 10.1038/s41598-020-75347-4 (PMC7589565; doi:10.1038/s41598-020-75347-4)
Supplement: Supplementary file 12 — Supplementary Information. [file 41598_2020_75347_MOESM12_ESM.docx]

**Visualization and quantification of dynamic intercellular coupling**

**in human embryonic stem cells using single cell sonoporation**

Zhenzhen Fan^1^, Xufeng Xue^2^, Jianping Fu^1,2^, and Cheri X. Deng^1,2,^*

^1^Department of Biomedical Engineering, University of Michigan, Ann Arbor, MI 48109, USA;

^2^Department of Mechanical Engineering, University of Michigan, Ann Arbor, MI 48109, USA;

*Correspondence: cxdeng@umich.edu

**Supplemental Figures**


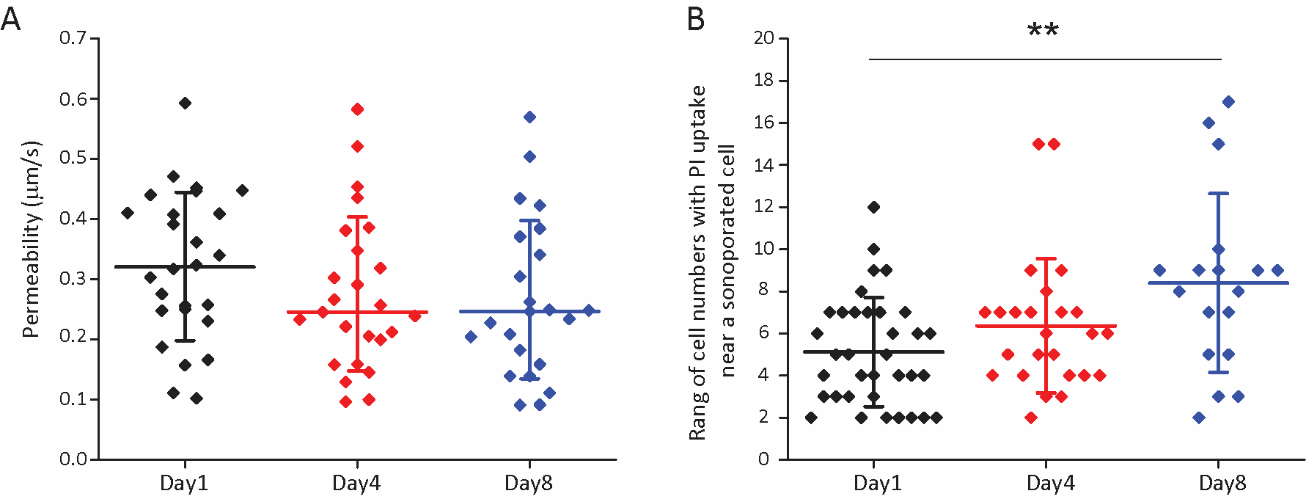


**Figure S1**: Permeability and range at different days of hESC culture. (A) GJ permeability and (B) Number of successive cells exhibited PI uptake resulted from PI loading in a sonoporated cell.


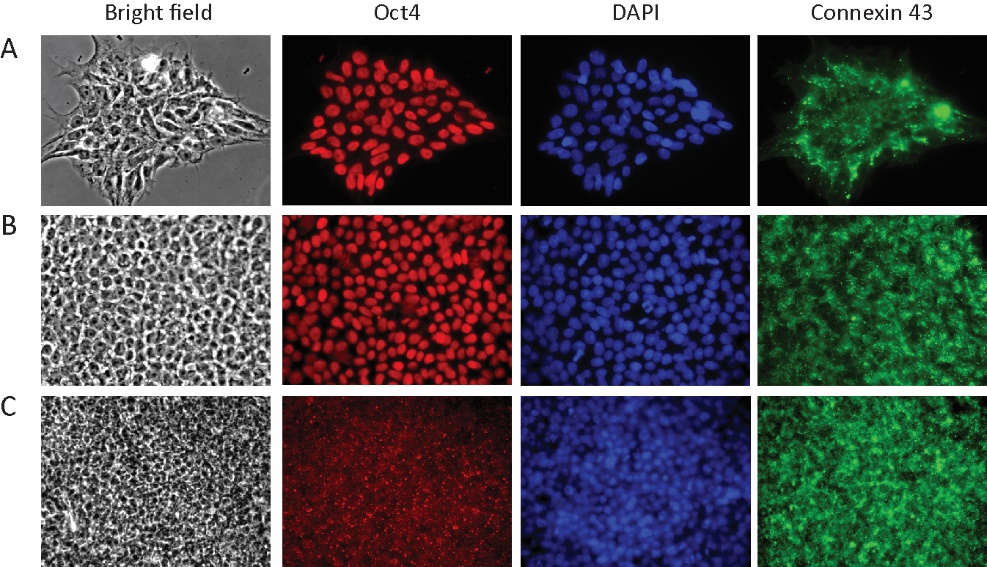


**Figure S2**: Immunostaining of connexin 43 for hESCs on day 1 (A), day 4 (B), and day 8 (C).


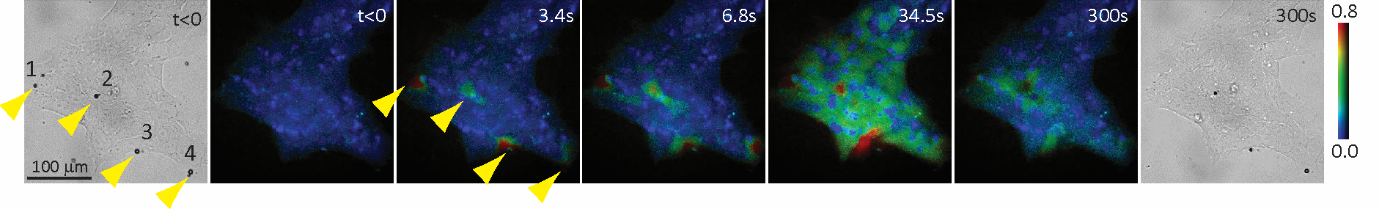


**Figure S3**: Sonoporation of multiple cells via cell attached microbubbles (arrows in the first bright field image) in a hESC colony induced multiple intercellular Ca^2+^ waves. The initial speed of wave propagation shown was 7.96, 7.25, 9.79 and 8.58µm/s for location 1 – 4 respectively.


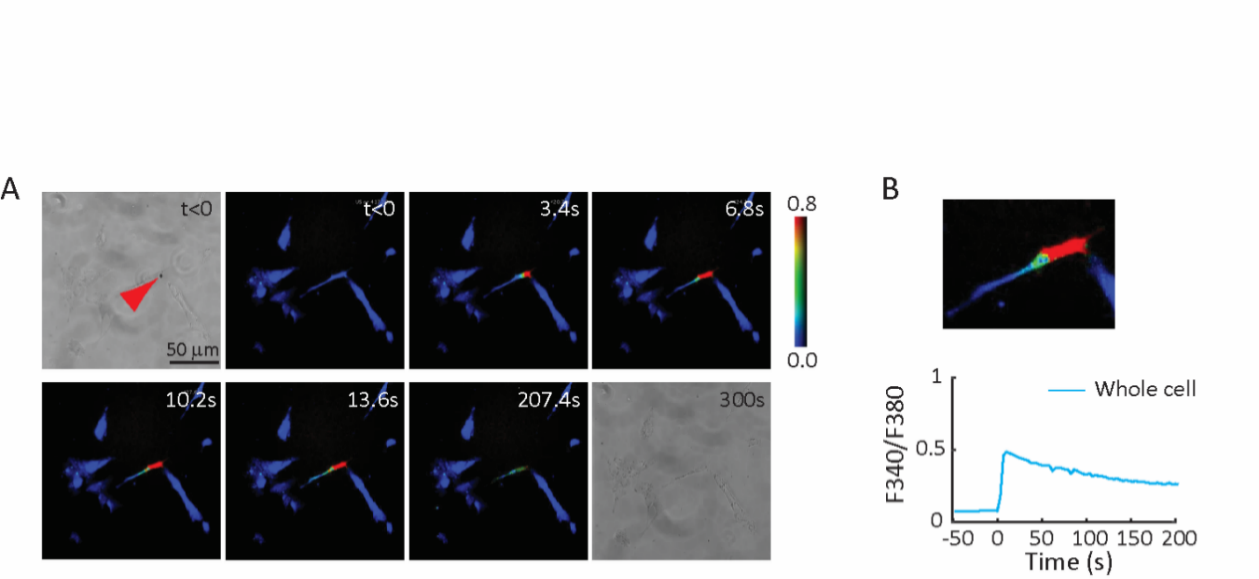


**Figure S4**: (A) Single cell sonoporation generated influx of Ca^2+^ and intracelullar Ca^2+^ wave in the sonoporated cell, but not in the surrounding cells that were not in direct contact with the sonoporated cells. Even at small distance, e.g. 3.2 µm, no intercellular Ca^2+^ wave was generated in the surrounding cells. (B) Fura2 fluorescence ratio image and plot showing intracellular Ca^2+^ concentration in the sonoporated cell.

**Supplemental Videos**

**Movie S1**: Video of the result in Figure 2, showing that sonoporation facilitated by cavitation of cell-targeted microbubble enabled dynamic visualization of single cell dye (PI) loading and dye transfer via gap junctions in hESCs.

**Movie S2**: Video of the result in Figure 3, showing that sonoporation allowed dye loading in multiple single cells and dye transfer via gap junctions in hESC colony.

**Movie S3**: Video of PI diffusion for the result in Figure 4, showing intracellular diffusion of PI and dye transfer via gap junction after sonoporation-induced dye loading in a single hESC.

**Movie S4**: Video of the results in Figure 5 showing that sonoporation allowed dynamic visualization of single cell dye loading and dye transfer via gap junctions in hESCs for the estimation of gap junction permeability.

**Movie S5**: Video of results in Figure 6, showing dye loading by sonoporation at multiple locations and dye transfer via gap junctions permits estimation of cell-cell permeability of hESCs.

**Movie S6**: Video of the results in Figure S2 showing sonoporation of multiple cells via microbubbles (arrows in the first bright field image) in a hESC colony induces multiple intercellular calcium waves.

**Movie S7:** Video of the result in Figure 7 showing that single cell sonoporation generated influx of calcium and intracellular calcium wave in the sonoporated cell, but not in the surrounding cells without direct contact with the sonoporated cells.

**Movie S8**: Video of result in Figure S3 showing that single cell sonoporation generated influx of calcium and intracelullar calcium wave in the sonoporated cell, but not in the surrounding cells that were not in direct contact with the sonoporated cell.

**Movie S9**: Video of Figure 8 showing an discontinued calcium wave in hESCs after single cell sonoporation in hESC colony. A single short ultrasound pulse (8µs, 0.4MPa) induced calcium influx in one cell, followed by discontinued intercellular calcium wave.

**Movie S10:** Video of a symmetric calcium wave in hESCs after single cell sonoporation in hESC colony as shown in Figure 9A. A single short ultrasound pulse (8µs, 0.4MPa) induced calcium influx in one cell, followed by discontinued intercellular calcium wave.

**Movie S11**: Video of an asymmetric calcium wave and calcium oscillation in hESC colony generated by single cell sonoporation as shown in Figure 9B. A single short ultrasound pulse (8µs, 0.4MPa) induced calcium influx in one cell, followed by discontinued intercellular calcium wave.
